# Strongyloides Hyperinfection Syndrome Triggered by a Single Dose of Dexamethasone Administered for Fetal Lung Development

**DOI:** 10.7759/cureus.46067

**Published:** 2023-09-27

**Authors:** John Meisenheimer, VII, Jaraad Dattadeen, Meredith Thomley, Sadaf Aslam

**Affiliations:** 1 Internal Medicine, University of South Florida, Tampa, USA; 2 Dermatology, University of South Florida, Tampa, USA

**Keywords:** strongyloides stercoralis, hypersensitivity pneumonitis (hp), protein-losing enteropathy, postpartum sepsis, postpartum complication, placental abruption, gram-negative bacteremia, strongyloides hyperinfection syndrome, paralytic ileus

## Abstract

Strongyloides hyperinfection syndrome is a rare manifestation caused by the *Strongyloides stercoralis* parasite and has mortality rates close to 90% if left untreated. Corticosteroids are commonly implicated as a trigger for hyperinfection syndrome in patients with Strongyloides autoinfection, and it has been suggested that even a single dose of corticosteroids can trigger hyperinfection syndrome. Here, we report a case of hyperinfection syndrome eight days after administering a single 8 mg dose of dexamethasone for fetal lung development before a late preterm, emergency cesarean section (C-section) delivery secondary to placental abruption.

Prior to the C-section, the patient had been exhibiting signs of autoinfection syndrome, cough, and abdominal pain, for several months. Following corticosteroid administration, she had sequelae of Strongyloides hyperinfection syndrome, including gram-negative bacteremia, undulating fevers, protein wasting enteropathy, and hypersensitivity pneumonitis. Sputum cultures were positive for Strongyloides, and after treatment with ivermectin and albendazole, the patient fully recovered.

Strongyloides hyperinfection syndrome is a documented consequence of short courses of corticosteroids. Still, this case is unique because the patient only received a single dose of corticosteroids before developing hyperinfection syndrome. Clinicians must recognize patients at risk for Strongyloides hyperinfection syndrome and understand the risks of administering corticosteroids to patients harboring the parasite.

## Introduction

Strongyloides hyperinfection syndrome is a rare manifestation caused by the *Strongyloides stercoralis* parasite and has mortality rates close to 90% if left untreated [1]. Corticosteroids are commonly implicated as a trigger for hyperinfection syndrome in patients with Strongyloides autoinfection, and it has been suggested that even a single dose of corticosteroids can trigger hyperinfection syndrome [1,2]. Here, we report a case of hyperinfection syndrome in a 23-year-old Nicaraguan immigrant eight days after administering a single, 8 mg dose of dexamethasone for fetal lung development before a late preterm, emergency cesarean section (C-section) delivery.

## Case presentation

A 23-year-old Nicaraguan immigrant female G5P0040 at 34 weeks gestation with a previous medical history of four prior miscarriages between 10 and 20 weeks was admitted for syncopal episodes, transaminitis, and starvation ketosis secondary to intractable vomiting. During the pregnancy, she was evaluated multiple times for dry cough, diffuse abdominal pain, nausea, and vomiting; symptoms began after consuming vegetables her friends brought from Honduras.

**Figure 1:**
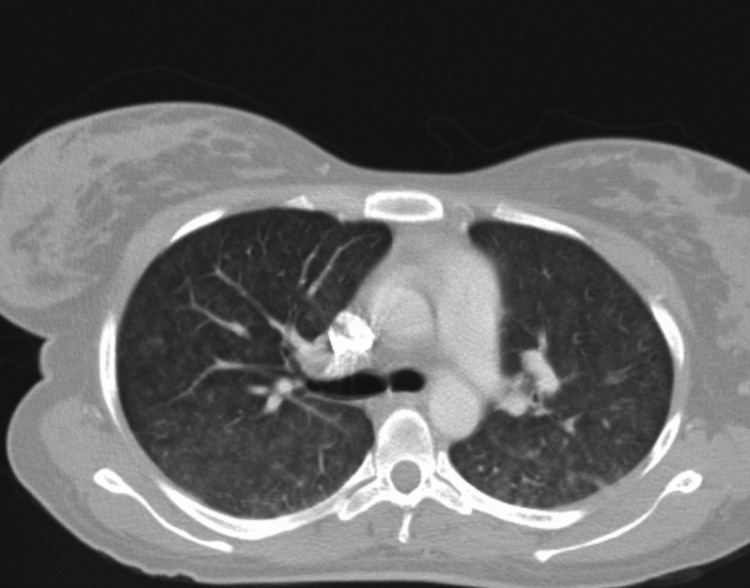
CT Pulmonary Angiography Computed tomography (CT) pulmonary angiography showing ground glass opacities consistent with hypersensitivity pneumonitis.

**Figure 2:**
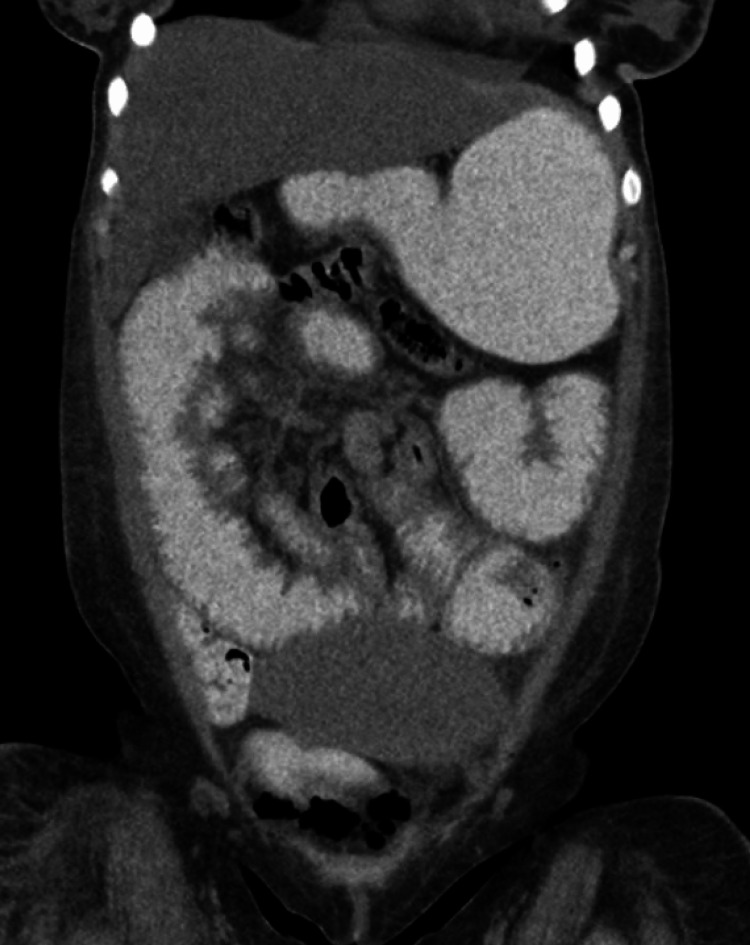
CT Abdomen with Oral Contrast Computed tomography (CT) abdomen with oral contrast showing a transition point and proximal dilated bowel with distal decompressed bowel, concerning intestinal obstruction.

**Figure 3:**
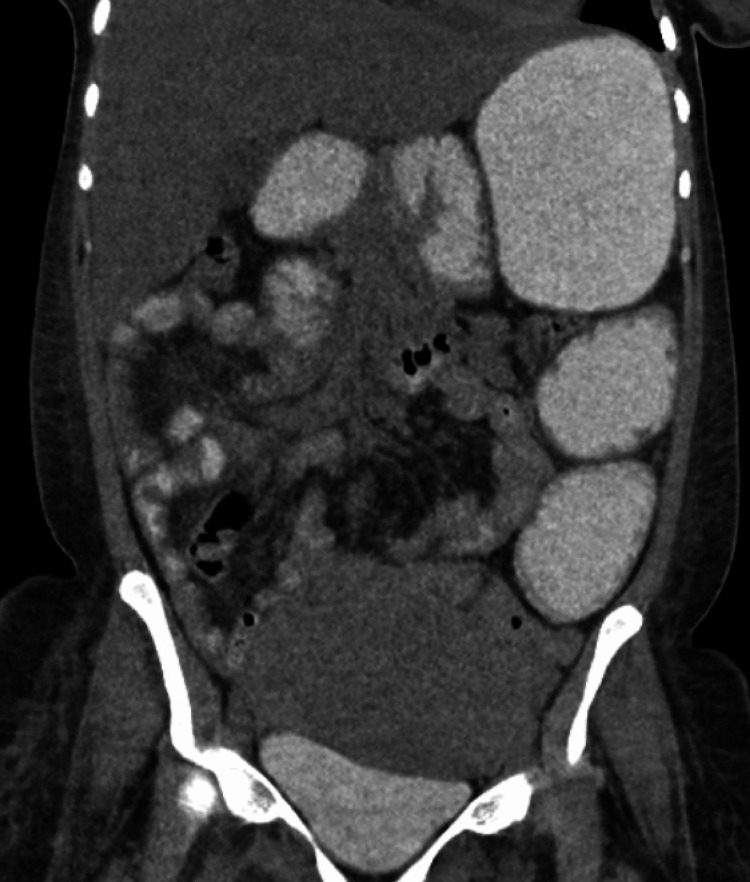
CT Abdomen with Oral Contrast Computed tomography (CT) abdomen with oral contrast showing delayed transit of contrast, making ileus more likely than obstruction.

At 35 weeks and two days, she had a placental abruption, was given 8 mg of dexamethasone (equivalent to 55 mg of prednisone), and underwent a C-section. The patient’s baby was healthy at birth, with no significant medical complications while hospitalized. Following the C-section, the patient experienced constipation and worsening abdominal pain. CT pulmonary angiography on post-op day two showed changes consistent with hypersensitivity pneumonitis (Figure 1). CT of the abdomen and pelvis with positive oral (PO) contrast at post-op day four showed possible obstruction (Figure 2), with repeat CT post-op day five confirming adynamic ileus (Figure 3). On post-op day eight, she developed sepsis with a fever up to 102.9⁰ F, tachypnea over 24 respirations per minute, and tachycardia over 160 beats per minute. Blood cultures were positive for Extended Spectrum Beta Lactamase (ESBL) *Escherichia coli*. Evaluation of the C-section wound showed a healing lesion without concern for infection. The patient also developed 3+ pitting edema with serum albumin levels of 1 g/dL. Doppler ultrasound showed no deep vein thrombosis (DVT). On chart review of labs collected before admission, it was noticed that she had a stool ova and parasite (O&P) collected a month prior as part of the workup for her persistent dry cough and abdominal pain during pregnancy, which was positive for Strongyloides but was not followed up on. Due to concerns for hyperinfection syndrome, ivermectin 400 mcg/kg once daily was started.

Over the following month, the patient had undulating fevers, up to 104°F, that responded to meropenem. Multiple blood cultures were taken and were negative. Induced sputum sample on post-op day 24 showed Strongyloides larva and fungal elements. Albendazole 400 mg twice daily was initiated with plans to continue treatment for 24 days. Abdominal pain continued throughout her admission but gradually improved following albendazole.

Post-op day 34, the patient had a negative stool O&P and ivermectin was discontinued. Follow-up stool O&P post-op day 38 was also negative. Immediately before discharge, the patient had a positive QuantiFERON gold test with radiographic findings negative for tuberculosis disease and resolution of her symptoms of cough and fevers with treatment for Strongyloides. Plans were made to treat her for latent tuberculosis following discharge. Serology was also positive for human T-cell lymphotropic viruses (HTLV1/2) antibodies. The patient was discharged on albendazole post-op day 44. She did not return for any scheduled follow-up appointments.

## Discussion

While *Strongyloides stercoralis* infection is uncommon in the US, with an estimated prevalence of 0-6%, patients from endemic locations can unknowingly harbor the parasite for many years, in a process called autoinfection [1,3,4]. Strongyloides hyperinfection is a rare and life-threatening complication of the *Strongyloides stercoralis* parasite, in which immunosuppression causes rapid parasite proliferation [4]. Our patient exhibited many symptoms seen with hyperinfection syndrome, including hypersensitivity pneumonitis, protein loss enteropathy leading to hypoalbuminemia and pitting edema, gram-negative bacteremia, and small bowel ileus [1].

In a PubMed search for articles containing the keywords “pregnancy” and “Strongyloides,” we found only one other reported case of hyperinfection syndrome during pregnancy. The patient had a placental abruption at 25 weeks gestational age and was given two doses of betamethasone 24 hours apart. Treatment with ivermectin was ineffective, and the patient died from complications of hyperinfection syndrome [5]. Interestingly, serology done on this patient was also positive for HTLV1 antibodies. HTLV1 has been implicated as a risk factor for hyperinfection syndrome due to Th2 immune response dampening, and coinfected individuals typically have a more severe and complicated course [6].

With an estimated 100 million people infected with Strongyloides worldwide, clinicians must be aware of this life-threatening complication and the risk of precipitating hyperinfection when giving corticosteroids for fetal lung development [5]. In fact, for patients who have spent extended time living in areas endemic to *Strongyloides stercoralis*, which includes most tropical and subtropical regions, the WHO is considering the evidence for the presumptive treatment of *Strongyloides stercoralis* with ivermectin before administration of corticosteroids [7]. Evidence for the safety of ivermectin during pregnancy is limited, and ivermectin is currently pregnancy category C [1,8]. Clinicians should consider the risk of hyperinfection syndrome versus the benefit to the fetus when administering corticosteroids for late preterm delivery in patients infected with *Strongyloides stercoralis*, even more so in patients coinfected with HTLV1.

## Conclusions

The case presented here represents one of two cases of Strongyloides hyperinfection syndrome following corticosteroids for fetal lung development reported in the literature. Unlike the prior case, the patient presented here received only a single dose of corticosteroids. As a rare manifestation of a common parasite worldwide, this case is important for practitioners in the US and other countries endemic for Strongyloides. Clinicians must be aware of the risks of administering corticosteroids to patients harboring the Strongyloides parasite.
